# Liquor Potassæ a Test for Diabetic Urine

**Published:** 1844-11-16

**Authors:** 


LIQUOR POTASSÆ A TEST FOR DIABETIC URINE.

Mr. Moore states, from experiments which he has made, that liquor potassæ, when boiled with diabetic urine, affords a reddish-brown precipitate, and a characteristic rich claret colour. Mr. Palmer, he says, was the first to notice this.—*Ibid.*

